# Bidirectional relationship of stress and affect with physical activity and healthy eating

**DOI:** 10.1111/bjhp.12355

**Published:** 2019-01-22

**Authors:** Dana Schultchen, Julia Reichenberger, Theresa Mittl, Tabea R. M. Weh, Joshua M. Smyth, Jens Blechert, Olga Pollatos

**Affiliations:** ^1^ Department of Clinical and Health Psychology Ulm University Germany; ^2^ Centre for Cognitive Neuroscience University of Salzburg Austria; ^3^ Department of Psychology University of Salzburg Austria; ^4^ Department of Biobehavioral Health and Medicine Pennsylvania State University State College Pennsylvania USA

**Keywords:** affect, ecological momentary assessment, healthy eating, physical activity, stress

## Abstract

**Objectives:**

Physical activity and healthy eating seem to be protective against experiencing stress and negative affect as well as increase positive affect. At the same time, previous studies showed that people reduce salutogenic behaviours such as physical activity and healthy eating in the face of stress and negative affect while increasing such behaviours in the context of positive affect. Due to daily fluctuations of these behaviours, the present study examined these relationships in daily life using ecological momentary assessment (EMA).

**Design and methods:**

Fifty‐one university students responded to six daily prompts during 7 days via smartphone‐based EMA. Items examined stress, emotional experience, physical activity duration, and healthy eating.

**Results:**

Higher stress and negative affect, as well as lower positive affect, were related to a reduction in subsequent physical activity. Higher physical activity levels, in turn, were associated with less subsequent stress and negative affect, as well as more positive affect. No such effects for stress and affect on healthy eating or vice versa were found.

**Conclusions:**

Engaging in physical activity is related to better mood and less stress/negative affect over the next several hours in daily life. Prevention efforts therefore may benefit by focusing on promoting physical activity, particularly when stress/negative affect is high to ‘break the cycle’ of inactivity, stress, and negative affect. Potential effects of healthy eating might be more subtle and characterized by interindividual differences or state effects.

**Statement of contribution**

***What is already known on this subject?***

- Physical activity can reduce stress as well as negative emotions and can enhance positive emotions.
- People tend to eat less healthy food during stressful times, and healthy eating can increase general health.
- Physical activity and healthy eating have been mostly assessed separately and through retrospective methods.

***What does this study add?***

- This is an EMA study investigating bidirectional effects of stress, emotions, and salutogenic behaviour.
- After physical activity, people felt less stressed/negative and more positive; feeling better and less stressed resulted in more physical activity.
- Healthy eating was not associated with stress or emotion level and vice versa.

## Background

Feeling stressed is a growing phenomenon of modern society (e.g., Cohen & Janicki‐Deverts, 2012; Hapke *et al*., 2013) and has become a primary health concern, especially in the population of students (e.g., Bayram & Bilgel, 2008; Beiter *et al*., 2015; Techniker Krankenkasse, 2013). It is associated with health‐related problems such as gastrointestinal, endocrine, and cardiovascular disease, as well as mental disorders like anxiety and depression (Cohen, Janicki‐Deverts, & Miller, 2007; Dimsdale, 2008; Gerber, Lindwall, Lindegård, Börjesson, & Jonsdottir, 2013; Misra & McKean, 2000; Mouchacca, Abbott, & Ball, 2013). In the light of these pervasive negative consequences, identifying potential salutogenic behaviours such as physical activity and healthy eating for the stress–health relationship seems necessary (e.g., Gerber & Pühse, 2009; Wienbergen & Hambrecht, 2013).

### Stress and physical activity – bidirectional relationships

Physical activity is defined as any movement of the body that results from skeletal muscles using energy (World Health Organization, 2010). The minimum of recommended activity standards (150 min of moderate physical activity, 75 min of vigorous physical activity per week, or a combination of both) is only reached by approximately 10% of adults (e.g., Tucker, Welk, & Beyler, 2011; World Health Organization, 2010). Physical inactivity, for example, sedentary behaviour, can contribute to serious physiological and psychological problems such as increased risk for obesity, metabolic syndrome, cancer, cardiovascular problems, and worse body satisfaction and self‐esteem (for a review, see Tremblay, Colley, Saunders, Healy, & Owen, 2010). In contrast, even a medium amount of daily physical activity can help to prevent as well as improve general physical and mental well‐being, including stress level (Fox, 1999; Penedo & Dahn, 2005). Hence, for prevention and intervention programmes, it is important to identify possible factors influencing the activity level – like stress and affect (Kahn *et al*., 2002) – that may be helpful in counteracting the negative consequences of physical inactivity (Bauman *et al*., 2012).

Previous studies have shown that both physical activity and stress mutually influence each other. During stressful periods, people tend to engage in activities that are less exhausting and avoid exercise probably due to a lack of time and self‐regulatory resources (e.g., Kouvonen *et al*., 2013; Nelson, Lust, Story, & Ehlinger, 2008; Sonnentag & Jelden, 2009; Teisala *et al*., 2014). Moreover, Stults‐Kolehmainen and Sinha (2014) reviewed studies showing that stress prospectively predicts a decrease in physical activity. The authors emphasized that instead of using physical activity to deal with stress, people often use unhealthy behaviours as an expression of emotion‐focused coping, including smoking, overeating, drinking, and a lack of exercise.

There is also an indication for the reverse direction of causality in that physical activity is associated with less stress. It was demonstrated that activity level is related to objective stress markers as well as subjective stress perception (e.g., Klaperski, von Dawans, Heinrichs, & Fuchs, 2013; Lippke, Wienert, Kuhlmann, Fink, & Hambrecht, 2015). Rimmele *et al*. (2009) found that regular physical activity is associated with lower objective indicators of stress reactivity (salivary cortisol) following a standardized stressor in sportsmen who exercise regularly compared to inactive controls with no exercise training experience. Moreover, similar findings have been found for other physical parameters (e.g., heart rate, cardiovascular recovery after stress exposure; Forcier *et al*., 2006; Jackson & Dishman, 2006; Teisala *et al*., 2014). In addition, similar patterns of results are seen when examining subjective stress level measured by questionnaires (e.g., Lippke *et al*., 2015; Lutz, Stults‐Kolehmainen, & Bartholomew, 2010; Nguyen‐Michel, Unger, Hamilton, & Spruijt‐Metz, 2006; Rueggeberg, Wrosch, & Miller, 2012). To sum up, most of the previous studies measured physical activity by questionnaires or objective markers and primarily tested between‐person associations. Thus, there is an inherent need to utilize methods that can examine dynamic, within‐person processes (for a review, see Kanning, Ebner‐Priemer, & Schlicht, 2013). Moreover, it is difficult to capture changes such as stress and affect through salutogenic behaviour using global recall/retrospection. As such, there is a great need for approaches such as ecological momentary assessment (EMA; Shiffman, Stone, & Hufford, 2008; Smyth & Heron, 2012) that can more directly assess within‐person processes along with physical activity and related behaviours.

### Stress and healthy eating – bidirectional relationships

Another important salutogenic behaviour with regard to prevention of (existing) disease and control of weight is healthy eating. In contrast to physical activity, there is no precise definition of healthy eating, but rather individuals variously define healthy eating based on a low amount of fat, natural/unprocessed food, and/or a balanced (nutrient) eating style (Falk, Sobal, Bisogni, Connors, & Devine, 2001). Earlier studies support the idea that stress alters eating behaviour regarding the overall calorie consumption (Oliver & Wardle, 1999; Torres & Nowson, 2007; Zellner *et al*., 2006). Furthermore, previous literature showed a change of the macronutrient composition during stressful times with an increase in the consumption of high‐fat, high‐sugar foods (Habhab, Sheldon, & Loeb, 2009; O'Connor, Jones, Conner, McMillan, & Ferguson, 2008) and a decrease in fruits and vegetables consumption (Cartwright *et al*., 2003; O'Connor *et al*., 2008; Oliver & Wardle, 1999). Moreover, people engage in more snacking behaviour when stressed in naturalistic and cross‐sectional studies (Cartwright *et al*., 2003; O'Connor *et al*., 2008; Oliver & Wardle, 1999; Zenk *et al*., 2014). In sum, stress appears generally associated with eating behaviour changes in an unhealthy, rather than healthy, direction. Although these studies have many strengths (e.g., often high internal validity and control in laboratory settings), they may have questionable ecological validity due to the non‐naturalistic nature of eating in laboratory settings (Robinson, Hardman, Halford, & Jones, 2015) and often cross‐sectional designs. EMA studies, by contrast, consider the daily fluctuations in stress and eating behaviour and minimize bias. O'Connor *et al*. (2008), for example, showed that the daily hassles increase unhealthy eating behaviour through a tendency towards high‐fat/high‐sugar snacks as well as a reduction of main meals and vegetables consumption. So far, there are only a few of these studies examining the frequency of snacking and changes in macronutrient composition as outcome measures of stress in EMA studies (Conner, Fitter, & Fletcher, 1999; Newman, O′Connor, & Conner, 2007; O'Connor *et al*., 2008; Zenk *et al*., 2014). Extending previous approaches, the current EMA study used a single item focusing on a generic measurement of healthy eating in order to comprehensively assess various aspects of eating behaviour. Studies suggest that individuals’ assessment of their healthy eating behaviour comprises judgements on a meal's macronutrient composition (Paquette, 2005), and appear to be relatively in line with dietary guidelines that lower levels of fat, sugar, and salt as well as higher levels of vegetables and fruits are viewed as healthy (for a review, see Paquette, 2005).

Again, there is also an indication for the reverse direction of causality, with healthy eating leading to improved bodily and mental health (Prasad, 1998). Therefore, we posit that healthy eating may decrease stress and serve as another key salutogenic behaviour for reducing stress. Alternatively, eating can be used in order to distract from stress, thereby alleviating perceived emotional stress (Macht, Haupt, & Ellgring, 2005). Earlier studies explored the reverse relationship mainly for affect changes after the consumption of certain food types, mainly carbohydrates, meaning that people feel better after consumption and reduce their stress level (Christensen & Pettijohn, 2001; Evers, Stok, & de Ridder, 2010; Spring, Chiodo, & Bowen, 1987; Sproesser, Schupp, & Renner, 2014; Taut, Renner, & Baban, 2012). Likewise, fruits and vegetables consumption seems to lead to higher subsequent positive affect (Conner, Brookie, Carr, Mainvil, & Vissers, 2017; Conner, Brookie, Richardson, & Polak, 2015; White, Horwath, & Conner, 2013).

### The present study

The present EMA study investigated the bidirectional relationships of stress with physical activity and of stress with healthy eating in daily life. As an additional goal, we considered the role of positive and negative affect, as stress and affect have frequently been intermingled and seem to have combined effects. Studies indicate that affect seems to have an influence on an individual's activity level (e.g., Cameron, Bertenshaw, & Sheeran, 2017; Herman *et al*., 2002; Schwerdtfeger, Eberhardt, Chmitorz, & Schaller, 2010; Wallbott, 1998) and eating behaviour (for an overview, see Cardi, Leppanen, & Treasure, 2015; Macht, 2008) and that salutogenic behaviours have a positive effect on affect (e.g., Conner *et al*., 2017; Dunton *et al*., 2014; Lathia, Sandstrom, Mascolo, & Rentfrow, 2017; White *et al*., 2013; Wichers *et al*., 2012).

Similar to previous research, we used a naturalistic and often intense academic stressor, the examination period, in an attempt to ensure a considerable stress load (von Haaren, Haertel, Stumpp, Hey, & Ebner‐Priemer, 2015). We tested four main hypotheses: First, it was hypothesized that physical activity is reduced subsequent to periods marked by higher stress and negative affect. Furthermore, we assumed that higher positive affect goes along with higher physical activity. Second, we expected a reverse effect, meaning that higher physical activity levels are related to subsequently reported less perceived stress/negative affect and more positive affect. Third, regarding the stress–healthy eating relationship, we expected that unhealthier eating is reported during times marked by higher stress, negative affect, and positive affect. Finally, the reverse direction, a relationship of healthy eating on stress and affect, was analysed, assuming that healthy eating is associated with subsequently less stress and negative affect as well as higher positive affect.

## Method

### Participants

We recruited 56 healthy students by email list announcement, notices, and word of mouth to participate in the EMA study ‘The effects of stress on eating behaviour and physical activity in daily life’. Due to smartphone device problems or overall low compliance (<50%), five participants were excluded for data analyses; apart from that, only complete EMA signals were used. In total, 51 students (11 men) with a mean age of 23.5 (*SD* = 2.6; range: 19–29) and a mean BMI of 22.3 kg/m² (*SD* = 3.2; range: 17.2–33.1) took part in the study. All procedures were conducted in accordance with the Declaration of Helsinki and with the approval from the institutional review board. Prior to testing, all participants were informed verbally and in writing about the study and signed an informed consent.

### EMA measures^1^

Ecological momentary assessment data were collected using the PsyDiary app (https://www.smarthealth.at/project/psydiary-2/). It was especially designed in collaboration with the Smart Health Check research group at the department of MultiMediaTechnology of the University of Applied Sciences. Questions for the PsyDiary app can be defined via LimeSurvey (Schmitz, 2015), and the app can be installed with Android and iOS devices. Participants were prompted six times per day over 8 days (including 1 day of training; data not used for the analyses) and completed affect and stress items and reported their physical activity level and eating behaviour. First, they reported how they felt in that moment on a visual analogue scale of continuous horizontal rating slider ranging from 0 to 100 (not at all–very much). Therefore, 12 different emotions, six positive (active, cheerful, enthusiastic, relaxed, calm, and awake) and six negative emotions (depressed, bored, irritated, dissatisfied with self, worried, and nervous/stressed), were presented randomly. Second, perceived stress was assessed with two items of the Perceived Stress Scale (PSS; Cohen, Kamarck, & Mermelstein, 1983; German version by Büssing, 2001): ‘Do you feel that you can cope with things’ and ‘Do you feel you′re on top of things’, rated from 0 (not at all) to 100 (very much); as well as the emotion item ‘nervous/stressed’. All items of the PANAS and PSS were adapted for the momentary use and assessed affect/stress similar to a recent study (Reichenberger *et al*., 2018).

Regarding physical activity, participants had to answer the question ‘How many minutes have you been physically active since the last signal so that you sweated or were out of breath’. In addition, physical activity was more specifically explained to participants as feeling out of breath or sweating as well as smaller daily activities (comparable with the study of Krug *et al*., 2013) in a manual they received at the beginning of the study. Participants estimated their physical activity between 0 and 160 min. In support of the validity of the physical activity question in the EMA, we calculated the relation between subjective physical activity and an objective assessment via actigraphy, revealing a significant correlation (see Appendix S1). Lastly, eating episodes since the last signal were reported and estimated with regard to their healthiness (0 = unhealthy; 100 = healthy) by asking participants ‘How would you consider your meal?’. Participants had the option to report up to three distinct eating episodes in between two prompts. In support of the validity of the healthy eating question in the EMA, naturalistically assessed healthy eating related to the consumptions of typically healthy considered food categories (e.g., fruits, vegetables) in the EMA and questionnaire‐based healthy eating (see Appendix S2).

### Procedure

The study was conducted in July and August 2016 during the examination period, which represents a natural stressor for students. Participants were invited to the laboratory and were informed about the purpose and procedure of the study. Next, they signed an informed consent to take part in the study. Afterwards, they completed demographic variables (e.g., age, gender), and a battery of psychological questionnaires on an online survey platform^2^. Then, participants were supervised for the installation of the PsyDiary app. Furthermore, important information for the use of the devices and the EMA items was provided and participants completed three practice trials in the presence of a research staff member to clarify their use. Moreover, participants received an app manual with a summary of the important information and contact details in case of any disturbances or questions. The first day was a practice day, and these data were not included in the analyses. Thus, participants became acquainted with the app and had the opportunity to ask questions arising in relation to the technique, app, and/or items. After this practice day, participants reported their affect, stress, physical activity, and eating behaviour during 7 days. More specifically, a signal‐contingent sampling was used, prompting participants every 2.5 hrs (9 a.m., 11.30 a.m., 2 p.m., 4.30 p.m., 7 p.m., 9.30 p.m.). Data segmentation was chosen to balance between obtaining sufficient data within the short study period, without overwhelming participants during their examination period. A 1‐hr time window was available to complete each assessment, with reminders every 10 min. If the participants did not reply within 1 hr, the current signal resulted in a missing value. In case of low compliance (meaning no answer of 50% signals daily) during this period, participants were contacted by the experimenter to remind them about signal completion. At the end of 1 week, students completed questions about reactivity and compliance and received monetary compensation of 25€ or three course credit points.

### Data analysis

In a first step, mean scores of the stress level, positive and negative affect, physical activity, and healthy eating were computed for each signal. The experienced stress level was determined by the mean of the reversed two PSS items as well as the affect item ‘nervous/stressed’, with higher scores indicating more stress. The averages of all six negative and positive affect items, respectively, were computed. Physical activity was used within every signal intraday. Lastly, the averaged healthy eating score across the three possible eating episodes for every signal was calculated.

In a second step, hierarchical linear models were calculated using HLM version 7 (Raudenbush, Bryk, Cheong, Congdon, & Du Toit, 2011) because of the nested, longitudinal data structure. Signals (Level 1) were nested within participants (Level 2). For testing the effects of stress/affect on subsequent physical activity/healthy eating, stress, negative affect, and positive affect from time *t*−1 were each modelled separately as Level 1 predictors of time *t* physical activity/healthy eating, respectively (in general, lagged scores were only calculated within day, not across days). These slopes represent unstandardized coefficients (β‐scores). At the same time, we controlled for autocorrelation by including physical activity/healthy eating from *t*−1 as predictors for physical activity/healthy eating at time *t*. For assessing the effects of physical activity/healthy eating on stress/affect, healthy eating and physical activity, respectively, were modelled as Level 1 predictors of stress, negative affect, and positive affect reported at signal *t*. At the same time, we controlled for autocorrelation by including stress/affect at time *t*−1 as predictors for stress/affect at time *t*. Consequently, at a given signal, participants had to a) report their momentary stress and affect level and b) their physical activity and healthy eating subsumed since the last signal. Level 1 predictors were person‐mean‐centred and intercepts as well as slopes allowed to vary randomly. Generally, all predictors and outcomes were analysed separately; however, if more than one predictor turned out significant, all significant predictors were entered simultaneously in a combined model in a next step. According to Nezlek (2012), standardizing Level 1 variables needs careful consideration: The recommended option, to z‐standardize Level 1 variables across the entire population of observations (here, all signals from all participants, ‘grand mean standardization’), differs from the approach of person‐mean centring. As our main aim was to examine how a person compares to themselves at different moments in time (e.g., during higher or lower stress as usual), we used person‐mean centring. However, again as recommended by Nezlek (2012), we did not use z‐standardization within persons; thus, coefficients for different predictors are not comparable to each other or to coefficients in other research. To account for the broad range in BMI and the potential contextual effects of weekend‐days versus weekdays, all analyses were separately recalculated using BMI (grand‐mean‐centred) and weekday (uncentred; coded 0 = weekday, 1 = weekend‐day) as control variable for the intercepts.

## Results

### EMA measures

Participants completed 84.2% (*SD* = 11.0%) of the prompted signals on average, reflecting good overall compliance (range: 52.4–100%). Table 1 shows mean and standard deviations of included variables.^3^ At the end of the study, when asked about reactivity, students did not think that the prompts influenced their physical activity (*M *=* *12.3, *SD* = 17.5) or their eating behaviour (*M *=* *20.7, *SD* = 21.2) on a scale from 0 (not at all) to 100 (very much).

**Table 1:** Descriptive statistics of variables measured in the present study

Variable	*M*	*SD*	*Min*	*Max*
Level 1				
Signals				
Stress (0–100)	31.2	17.6	0.00	89.0
Positive affect (0–100)	40.0	17.7	0.00	100
Negative affect (0–100)	14.5	12.9	0.00	63.5
Physical activity (0–160)	7.20	18.6	0.00	160
Health eating (0–100)	56.3	25.7	0.00	100

Note

*M *= mean; *SD* = standard deviation; Level 1 = all occasions within a participant.

### Stress and affect relating to subsequent physical activity

**Figure 1:**
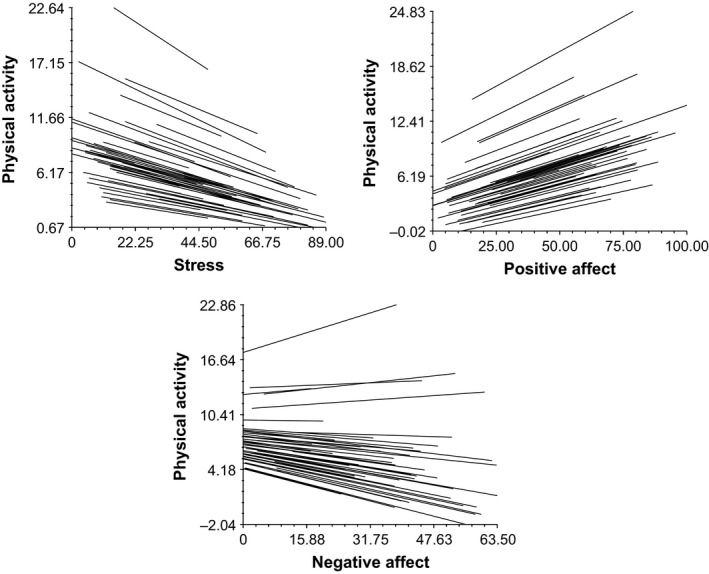
Stress as well as positive and negative affect (uncentred, at *t*−1) is related to physical activity (*t*) with each individual displayed separately.

As can be seen in Figure 1, higher stress significantly related to lower self‐reported physical activity (β_10_ = −.094, *SE* = .036, *p* = .012) over the next few hours, controlling for physical activity at time *t*−1 (β_20_ = .041, *SE* = .034, *p* = .231). Similarly, higher negative affect was marginally associated with lower physical activity (β_10_ = −.073, *SE* = .038, *p* = .057), controlling for physical activity at time *t*−1 (β_20_ = .044, *SE* = .033, *p* = .198), and higher positive affect related to higher physical activity (β_10_ = .090, *SE* = .027, *p* = .001), controlling for physical activity at time *t*−1 (β_20_ = .034, *SE* = .034, *p* = .325). The simultaneous prediction revealed only a significant result of positive affect (stress: β_30_ = −.077, *SE* = .056, *p* = .177; positive affect: β_20_ = .067, *SE* = .029, *p* = .027; negative affect: β_10_= .040, *SE* = .058, *p* = .489), controlling for physical activity at time *t*−1 (β_40_ = .035, *SE* = .033, *p* = .298).

### Physical activity relating to subsequent stress and affect

**Figure 2:**
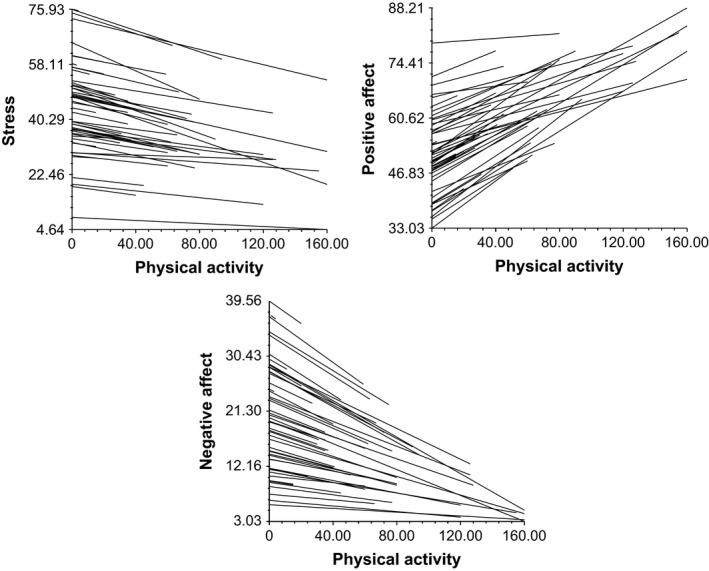
Physical activity (uncentred, at *t*) is related to stress as well as positive and negative affect (*t*) with each individual displayed separately.

As can be seen in Figure 2, more physical activity was associated with less subsequent stress (β_10_ = −.104, *SE* = .021, *p *< .001) when controlling for stress level at time *t*−1, which was a highly significant predictor for stress at *t* itself (β_20_= .393, *SE* = .036, *p *<* *.001). Positive affect at time *t*−1 was a highly significant predictor for positive affect at *t* (β_20_ = .308, SE = .031, *p *<* *.001). Meanwhile, more physical activity was associated with greater subsequent positive affect simultaneously controlling for positive affect the signal before (β_10_ = .193, *SE* = .029, *p *<* *.001). Likewise, negative affect at time *t*−1 related to subsequent negative affect at *t* (β_20_ = .337, *SE* = .033, *p *<* *.001). In addition, less physical activity was associated with more subsequent negative affect controlling for negative affect the signal before (β_10_ = −.099, *SE* = .015, *p *<* *.001).

### Stress and affect predicting healthy eating

Stress at the prior time point did not significantly relate to reported healthy eating (β_10_ = .043, *SE* = .108, *p* = .690), neither did healthy eating at time *t*−1 (β_20_ = −.023, *SE* = .055, *p* = .680). Neither did negative affect (β_10_ = −.012, *SE* = .151, *p* = .938), controlling for healthy eating at time *t*−1 (β_20_ = −.046, *SE* = .052, *p* = .385), nor positive affect (β_10_ = .002, *SE* = .096, *p* = .980), controlling for healthy eating at time *t*−1 (β_20_ = −.022, *SE* = .054, *p* = .690), relate to subsequent healthy eating.

### Healthy eating predicting stress and affect

Healthy eating was not significantly associated with subjectively reported stress (β_10_ = −.011, *SE* = .018, *p* = .541), controlling for stress at time *t*−1 (β_20_ = .428, *SE* = .045, *p* < .001). Neither did healthy eating significantly relate to negative affect (β_10_ = −.006, *SE* = .012, *p* = .645), controlling for negative affect at time *t*−1 (β_20_ = .339, *SE* = .038, *p* < .001), or positive affect (β_10_ = .026, *SE* = .020, *p* = .206), controlling for positive affect at time *t*−1 (β_20_ = .366, *SE* = .035, *p* < .001).^1^

## Discussion

The purpose of the present study was to examine bidirectional effects of stress and affect on physical activity and healthy eating in daily life, using EMA. Our results showed that higher stress/negative affect was associated with less subsequent physical activity, and more physical activity related to lower subsequent stress/negative affect levels. Likewise, stronger positive affect related to more physical activity, and more physical activity was associated with higher subsequent positive affect. Contrary to our predictions, stress and affect were unrelated to unhealthy eating, and healthy eating was unrelated to subsequent stress/affect levels.

### Stress/affect relating to subsequent physical activity

In line with previous research using between‐person designs (e.g., Mäkinen *et al*., 2010; Nelson *et al*., 2008; Stults‐Kolehmainen & Sinha, 2014), physical activity was lower shortly after stressful times. As physical activity can be seen as an effortful activity, less self‐regulatory resources, time constraints, or low motivation during stressful times might account for this effect (Conroy, Hyde, Doerksen, & Ribeiro, 2010; Sonnentag & Jelden, 2009). In accordance, a lack of time and exhaustion – potential side effects of stress – have been found to be major contributors of physical inactivity (Rao *et al*., 2012).

In addition, our results indicate that experiencing higher negative affect was associated with lower activity levels. Studies investigating affect before an exercise programme found that subjects with higher negative affect showed lower programme satisfaction and quicker dropout (Hamid, 1990; Herman *et al*., 2002). Moreover, affect levels of overweight subjects in the morning predicted the exercise level during the day, in that those with a more negative mood in the morning were less likely to engage in exercise during the day (Carels, Coit, Young, & Berger, 2007). However, previous research revealed mixed results, also indicating the reverse effect in that negative affect can increase low or moderate activity (Schwerdtfeger *et al*., 2010; Wallbott, 1998). In contrast to our study, the study by Schwerdtfeger *et al*. (2010) assessed objective physical activity over 12 hrs and future research should compare diverging findings examining subjective and objective measures over a longer period.

Further, our results showed that positive affect was associated with higher subsequent physical activity. This is complementary to experimental studies showing that induced positive affect can enhance physical activity (Peterson *et al*., 2012). A study by Cameron *et al*. (2017) suggested that an increase in physical activity could be due to the effects of positive affect on several physical activity goals: Positive affect increased the number and duration of physical activity episodes planned for the subsequent days and led to increased accessibility of physical activity goals as well as more activity during the examination period.

### Physical activity relating to subsequent stress and affect

Self‐reported recent physical activity was associated with lower subjective stress levels, even after accounting for the autocorrelation in stress. Consistent with between‐person research showing that exercise can have a stress‐reducing effect (e.g., Gerber *et al*., 2014), we found that even low/moderate daily activity was associated with significantly lower stress levels. Similarly, negative affect was lower and positive affect higher, following physical activity, further emphasizing the influence of activity on affect (e.g., Dunton *et al*., 2014; Lathia *et al*., 2017; Wichers *et al*., 2012). It should be noted that although previous studies found increased positive affect after physical activity, for negative affect, evidence is mixed: Studies indicate that a higher activity level is associated with lower negative affect (Dunton *et al*., 2014) or no effect of physical activity on negative affect (Wichers *et al*., 2012). Nevertheless, the affect results are mostly in accordance with, and extend, previous research in daily life (e.g., Giacobbi, Hausenblas, & Frye, 2005; Kanning & Schlicht, 2010). Potential mechanisms for the association between physical activity and stress/affect might be similar to exercising, which has been shown to alter neurotransmitter levels, for example, increasing dopamine, serotonin, and endorphins (e.g., Meeusen & De Meirleir, 1995; Ruscheweyh *et al*., 2011; Sutoo & Akiyama, 2003; Winter *et al*., 2007), thereby increasing positive affect and decreasing negative affect. In addition, affect changes after exercising might relate to self‐efficacy (Rhodes & Kates, 2015), in turn enhancing momentary affect (Pannicke, Reichenberger, Schultchen, Pollatos, & Blechert, submitted). Lastly, lower negative affect/stress and higher positive affect after physical activity might have motivational properties, thereby aiding in maintaining involvement in physical activity. Nevertheless, a previous review found effects on future physical activity behaviour only via affect changes during, but not after, exercising (Rhodes & Kates, 2015). Thus, future studies might profit from a more fine‐grained temporal resolution of affect ratings directly before, during, and after physical activity.

### No relation of stress/affect with subsequent healthy eating

Contrary to previous research and our predictions, the current results did not show any significant associations between stress/affect and healthy eating. It is possible, however, that our decision to focus on healthy eating might have overlooked several other important effects of stress/affect on eating behaviour (e.g., increased/decreased food intake; alterations in macronutrient composition; Evers *et al*., 2010; O'Connor *et al*., 2008; Sproesser *et al*., 2014; Taut *et al*., 2012; Torres & Nowson, 2007). Nevertheless, previous research suggested that the amount as well as the macronutrient composition of consumed foods is considered during meal healthiness assessment (Paquette, 2005). Likewise, previous research suggested that an individual's definition of healthy eating can be guided by one predominant theme or a set of multiple themes (e.g., eating in order to control weight, prevent diseases; eating low fat; Falk *et al*., 2001), with a complex diversity of considered aspects (e.g., aspects of nutrients; production of food; ways of eating; Bisogni, Jastran, Seligson, & Thompson, 2012). Hence, a uniform definition of healthy eating would be an important issue for future research. Consequently, our simple multidimensional assessment of healthy eating in this study may thus not have been optimal. Furthermore, previous studies suggested meaningful differences between individuals with regard to eating styles and sociodemographic variables, as hypothesized by Greeno and Wing (1994) in their individual differences approach.

### No relation of healthy eating with subsequent stress/affect

Also unexpectedly, healthy eating did not relate to subsequent stress/affect. Thus, global/general reports of healthy eating in our study might not reflect a salutogenic behaviour related to stress and affect directly: Previous research suggests that fruits and vegetables (i.e., healthy foods) can increase positive affect and reduce negative affect (Conner *et al*., 2015; White *et al*., 2013) pointing to different patterns depending on the specific type of food. In contrast, other research proposed that eating highly palatable (mostly unhealthy) foods as an emotion regulation strategy can reduce negative affect (e.g., Macht, 2008). However, as stated above, such palatable, high‐fat/high‐sugar foods might not be equivalent to what our participants considered unhealthy. Conversely, assuming that unhealthy eating might reduce stress/negative affect (negative reinforcement) does not directly translate into healthy eating increasing positive affect (positive reinforcement). That is, healthy eating might not be directly related to an instant relief in affect/stress: It is possible that healthy eating impacts well‐being with some delay, especially when physical consequences are its main driver (e.g., disease prevention). In addition, qualitative research has reported that healthy eating can be seen as pleasurable, as it reduces guilt and worry, but it is also seen as boring, not tasty, and not satisfying (Bisogni *et al*., 2012). One could speculate that these different associations vary within person with regard to food type or eating occasion, making the investigation of contextual and meal type factors for healthy versus unhealthy eating episodes a promising aim for future research.

### Limitations

First, the present study assessed stress levels six times a day and explicitly sampled during an examination period of the recruited students in order to capture naturalistic stress experiences; other sampling strategies might also be informative (see Smyth, Juth, Ma, & Sliwinski, 2017) to further generalize findings to varying stressors or non‐stressful life periods. Second, the choice of data segmentation and related lags (2.5 hrs) might influence our results and limit generalizability to longer or shorter time frames. Thus, more clearly documenting and testing the effects of physical activity/healthy eating and stress/affect across different time frames for both directional associations may be important (e.g., timing may differ in one direction from the other in a bidirectional association). Third, an inclusion of contextual effects of physical activity and healthy eating (e.g., environmental, situational, social/interpersonal factors) seems important (e.g., see Wichers *et al*., 2012) and might complement future research. Fourth, we recruited a sample comprising mainly female students, who might exhibit a healthier lifestyle in general, restricting generalizability. Future research might profit from a larger and more diverse sample with regard to employment (and related working hours), disturbed eating behaviours, or fitness level.

Fifth, one important limitation of our study is the reliance on subjectively reported variables: Stress was self‐reported and future research might profit from additional objective indicators of stress levels (e.g., cortisol levels or heart rate; Klaperski *et al*., 2013) and especially a combination of subjective and objective methods (Kanning *et al*., 2013). Similarly, the estimation of healthy eating was done by the participants, potentially biasing our healthy eating variable. Stevenson, Doherty, Barnett, Muldoon, and Trew (2007) found that participants reported an unbalanced view of healthy eating in that mostly fruits and vegetables were seen as healthy, while fatty, salty, and sweet foods were considered unhealthy. Also, we did not assess participants’ knowledge about food healthiness and which aspects (e.g., calorie content) they considered. In order to corroborate the current results, objective assessments of eating behaviour (e.g., electromyography‐based chewing or swallowing activity or doubly labelled water method; e.g., see Blechert *et al*., 2017) might aid in obtaining a rough amount of calorie intake as one approach to healthy eating. To measure healthy eating via the amount of intake of different food categories (e.g., vegetables vs. sweets), assessing pictures of the participants’ foods or detailed food diaries could aid in more objectively clustering foods in healthy versus unhealthy. Still, as discussed above, the approach to define healthy eating might be manifold and a more fine‐grained definition might be needed in future research. Likewise, physical activity was assessed subjectively implying that participants exhibit awareness for their physical activity which might be retrospectively biased. Thus, objective assessments of physical activity via fitness tracker, heart rate monitoring devices, etc., might be of interest in future research. In the present study, subjective physical activity correlated significantly with an objective assessment via actigraphy (see Appendix S1). However, subjective and objective assessment of physical activity might not always be in accordance with various between‐person influences (Kapteyn *et al*., 2018; Prince *et al*., 2008). Therefore, research is needed examining the divergence and coherence between subjective and objective assessment of physical activity and the effects with stress and emotions (Pannicke *et al*., submitted). Nevertheless, the current study mainly relied on within‐person variation in physical activity/healthy eating in relation to affect/stress, implying that the exact level/height of physical activity and healthy eating might not be as necessary to obtain.

### Health implications

Despite the limitations, the current results have relevance for the development and implementation of prevention and intervention programmes. These data support the emerging evidence and view that less stress/negative affect as well as high positive affect is associated with engagement in healthy behaviours, especially physical activity in everyday life. Because of the high prevalence of stress and its detrimental effects (e.g., Beiter *et al*., 2015; Cohen & Janicki‐Deverts, 2012; Gerber *et al*., 2013; Hapke *et al*., 2013; Mouchacca *et al*., 2013), stress reduction interventions might not only directly influence health behaviours positively, but may also do so for long‐term health outcomes such as BMI. Closing the circle, health promotion activities might comprehensively target reducing stress/negative affect as well as enhancing positive affect and physical activity. Instead of intensive exercising, promoting more moderate physical activity (e.g., taking stairs instead of an elevator) might be sufficient for short‐term stress level reductions and could more easily be integrated into daily life.

## Funding

This work was funded by the Peer‐Mentoring Team‐Program (Line A) of the German Psychology Society (DGPs, section Health Psychology). In addition, this work was supported by the European Research Council (ERC) under the European Union's Horizon 2020 research and innovation programme (ERC‐StG‐2014 639445 NewEat) and the Austrian Science Fund (FWF): [I 02130‐B27].

## Conflict of interest

The authors declare no conflict of interest.

## Supporting information


**Appendix S1.** Physical activity.
**Appendix S2.** Healthy eating.

## Footnotes

1 Other data of the study have been reported in Reichenberger *et al*. (2018).

2 Other measurements that have been assessed (e.g., heartbeat perception task) are not of relevance for the current study.

3 Within‐person reliability was assessed using variance decomposition technique (see Shrout & Lane, 2012). Within‐person reliability was moderate for positive affect (Rc = .774), negative affect (Rc = .692), and stress (Rc = .747) according to Shrout (1998).
