# Supplementary material for: Bidirectional relationship of stress and affect with physical activity and healthy eating
Source: Br J Health Psychol. 2019 Jan 22;24(2):315–33. doi: 10.1111/bjhp.12355 (PMC6767465; doi:10.1111/bjhp.12355)
Supplement: Supplementary file 1 — Appendix S1. Physical activity. Appendix S2. Healthy eating. [file BJHP-24-315-s001.docx]

**Additional supporting information**

**Appendix S1: Physical activity**

Physical activity was subjectively assessed via the diary question “How many minutes have you been physically active since the last signal so that you sweated or were out of breath”, answered on a continuous rating slider ranging from 0 to 160 minutes.

Meanwhile objective physical activity was assessed via a combined heart rate and movement sensor (Actiheart, CamNtech Ltd, Cambridgeshire, UK). The device determines METs (metabolic equivalents of tasks), classified in sedentary behaviour, light, moderate or vigorous physical activity. To support the validity of the subjective physical activity assessment, subjective physical activity was predicted by light/moderate physica activity as well as an overall mean MET during the inter-beep interval.

Subjective physical activity was significantly predicted by overall objective physical activity (β_10_ = 13.16, SE = 2.65, *p* < .001), as well as light/moderate objective physical activity (β_10_ = .191, SE = .046, *p* < .001).

**Appendix S2: Healthy eating**

Healthy eating was subjectively assessed via the diary question “How would you consider your meal?”, answered on a continuous rating slider from 0 (=unhealthy) to 100 (=healthy).

Meanwhile, we obtained rough estimates of different food categories by asking participants to indicate to which amount they have eaten the following food-categories within every eating episode: sweets, carbohydrates, fatty foods (pizza, burger), salty snacks (crisps, pretzels), fruits and vegetables, from 0 (=nothing) to 100 (=very much). Indeed, results show that the subjective healthy eating assessment is positively associated with the healthy food categories, fruits (β_10_ = .365, *p* < .001) and vegetables (β_10_ = .307, *p* < .001), and negatively associated with the unhealthy food categories, sweets (β_10_ = -.611, *p* < .001), fatty foods (β_10_ = -.385, *p* < .001) and salty snacks (β_10_ = -.512, *p* < .001). Carbohydrates can be classified as neither healthy nor unhealthy and hence, no correlation was found (β_10_ = -.032, *p* = .359).

In addition we obtained a questionnaire score that assesses health promoting eating behaviors before the start of the study. Therefore, we used the subscale of the German “Fragebogen zur Erfassung des Gesundheitsverhaltens” (english: Questionnaire on Health Behavior; Dlugosch & Krieger, 1995). The questionnaire score was the Level 2 predictor, group-centered, predicting healthy eating (Level 1). Questionnaire-based healthy eating was associated with daily healthy eating (β_01_ = 10.98, *p* = .006).

Cambridge Neurotechnology Ltd (2010). The Actiheart User Manual (v. 4.0.35). Cambridge Neurotechnology Ltd: Cambridgeshire, United Kingdom.

Dlugosch, G.E. und Krieger, W. (1995). *Der Fragebogen zur Erfassung des Gesundheitsverhaltens (FEG)*. Frankfurt: Harcourt Test Gesellschaft.
